# Bioactive Compounds from Artichoke and Application Potential

**DOI:** 10.17113/ftb.61.03.23.8038

**Published:** 2023-09

**Authors:** Thais Feiden, Eunice Valduga, Jamile Zeni, Juliana Steffens

**Affiliations:** Food Engineering Department, Universidade Regional Integrada do Alto Uruguai e das Missões (URI), Erechim, Av. Sete de Setembro 1621, 99709-910 Erechim, RS, Brazil

**Keywords:** enzymes, polyphenols, antioxidant, methods of extraction, purification, bioactivities, applications

## Abstract

*Cynara cardunculus* L. var. *scolymus,* known as the artichoke, originated in the Mediterranean region and is now cultivated in several countries. The artichoke has leaves, a stem, and a head, also called a floral capitulum, covered with green and pointed bracts. It is rich in polyphenols, flavonoids, anthocyanins, phenolic compounds, inulin, coumarins, terpenes, dietary fibre, enzymes, polysaccharides, minerals and vitamins, and therefore has a wide range of uses, including in the food industry, medicine and biofuels. Several studies have shown that artichokes have properties such as antioxidant, anti-inflammatory, antimicrobial, anticancer, hypocholesterolaemic, anti-HIV, cardioprotective, hepatoprotective and lipid-lowering effects. The aim of this study is to provide a literature review on the phytochemical composition, bioactivity and applications, focusing on the methods of extraction, purification and concentration of enzymes present in artichoke.

## INTRODUCTION

*Cynara* is a small genus in the family *Asteraceae* with eight species and four subspecies, including the thistle (*Cynara cardunculus* L.), all native to the Mediterranean region (*1*, *2*). The thistle has three botanical varieties: the artichoke (*C. cardunculus* L. var. *scolymus*), the cultivated or leafy thistle (*C. cardunculus* L. var. *altilis*) and the wild thistle (*C. cardunculus* L. var. *sylvestris*) (*3*–*5*). According to the report of the Food and Agriculture Organization of the United Nations (*6*), the main producing countries are Italy, Egypt and Spain, which produce about 367 080, 308 844 and 196 970 tonnes per year, respectively (*7*–*9*).

*C. cardunculus* L. and its varieties are a rich source of a wide range of bioactive compounds, and the chemical composition is also quite variable among the different classes (*10*, *11*). Artichoke can be considered as a functional or nutraceutical food because it contains bioactive phytoconstituents such as polyphenols (phenolic acids, chlorogenic, caffeic, dicaffeoylquinic and ferulic acids), flavones (apigenin and luteolin) and their glycosides (apigenin-7-*O*-glucoside and cinnaroside), anthocyanins (cyanidin 3,5-diglucoside, cyanidin 3-glucoside, cyanidin 3,5-malonyldiglucoside, cyanidin 3-(3”-malonyl) glucoside and cyanidin 3-(6”-malonyl-glucoside), terpenoids (mono-, sesqui- and triterpenes), saturated (palmitic and stearic) and unsaturated (linoleic and oleic acid) fatty acids, carbohydrate polymers (inulin and pectin) (*1*, *12*, *13*), aspartic endopeptidases (EC 3.4.23) (cardosins/cyprosins or cynarase (cardosin A and B, cynarase A, B and C), polyphenol oxidase (EC 1.14.18.1) and peroxidase (EC 1.11.1.7) (*3*, *14*–*19*), minerals and vitamins (vitamin C, folates, biotin, niacin and pyridoxine) (*20*, *21*), among others, with potential health benefits (*22*, *23*). Thus, all parts of the plant have a wide range of uses (*12*, *24*–*27*). Biological activities include lipid-lowering, antioxidant, anti-inflammatory (*28*–*30*), hepatoprotective, cardioprotective, anticancer, antimicrobial and anti-HIV activities (*3*, *4*).

This paper presents a literature review of artichoke (*C. cardunculus* L.), its phytochemical composition, bioactivity and industrial applications, as well as a compilation of extraction methods, purification and concentration of enzymes present in artichoke.

## ARTICHOKE

The artichoke is native to the Mediterranean basin and adapts to different soil types and climatic conditions (*5*). Its origin and domestication may be in Sicily (*31*) in the XV century, from where it spread to Campania and Tuscany in Italy. By the beginning of the XVI century, it was cultivated in all Mediterranean countries, Central Europe and North America (*4*). At the beginning of the XX century, the artichoke was introduced to Brazil by European immigrants, especially Italians (*32*).

The artichoke has a whitish stem, large, lanceolate, fleshy, light green, pubescent leaves, and a scalloped appearance (*33*). The leaves can grow 50 to 200 cm long (*3*) and the plant can grow up to 150 to 180 cm tall (*34*). The flowers are violet-blue and large, look like small pine cones and are 7 to 10 cm in diameter. They form the inflorescence from the inner bracts (*17*), which consist of styles and stigmas (*4*, *35*), the bottom or heart being the edible part of the plant (*3*, *17*).

The number of flowers each plant produces varies. It produces one primary head and 4 to 20 secondary and tertiary flower buds per year (*3*). The heads or globes produced by the artichoke vary in size; the primary head is the largest and forms at the apex of the central stem, while the secondary and tertiary heads are smaller and develop on the branches (*20*).

Harvest is done manually by cutting the stem to a length of 20 to 30 cm. The ideal harvest time is when the buds have fleshy and adherent bracts (*36*). After the end of production, the aerial part of the dry plant is cut off at ground level, and in autumn the plant sprouts new shoots so that the cycle restarts again (*37*), with an average production period of 6 years (*38*).

Artichoke heads are consumed as fresh, preserved or frozen vegetables (*10*, *39*). A large amount of by-products (80*–*85 % of the total biomass) is produced during cultivation and industrial processing, with leaves, outer bracts, stalk, seeds and roots discarded (*5*, *17*, *40*). The stem can be separated from this total and used for consumption if properly processed (*20*).

By-products can also be used for the production of food additives and nutraceuticals (*17*, *40*), bioactive compounds such as polyphenols and inulin (*10*), in green chemistry for the production of cellulose, biofuels (*3*) and as fodder (especially stalks and leaves). In addition, they contain enzymes (proteases) that are used in the coagulation of milk (*20*). The root has a high sugar amount of about 25 % and an inulin content of 89.4 % (*3*), and the outer bracts have a higher inulin content than the stalk. The inulin content varies depending on the composition of the by-product, the subspecies of artichoke, its geographical origin and the time of harvest (*41*).

### Bioactive compounds

Artichoke heads contain, on dry mass basis, about 66.3 % total carbohydrates, 19.6 % protein, 2.0 % crude fat, 8.6 % ash (*20*, *42*) and 3.5 % fibre (*43*), and on fresh mass basis 14–19 mg/100 g vitamin C (*44*), folates and the B vitamin complex (biotin, niacin and pyridoxine) (*20*, *21*). Stalks, bracts and roots contain inulin, a soluble fibre (*5*, *41*, *45*). The artichoke is also a source of minerals such as potassium, calcium, sodium, magnesium, phosphorus, iron, copper and manganese (*43*, *46*).

The parts of the artichoke (stem, leaves, bracts and flowers) contain a number of primary and secondary metabolites, such as dietary fibre, polyphenols, flavonoids and terpenoids (*11*). Due to the presence of these compounds, it has beneficial properties for health (*3*, *5*, *47*).

The plant also contains sesquiterpene lactones, compounds responsible for 80 % of the bitter taste of artichoke leaves (*48*) and other plant tissues (*49*). Cinnaropicrin is the most important sesquiterpene lactone in *C. cardunculus* (*50*).

The by-products of the artichoke (stalk, leaves and bracts) are a source of pectin, which is widely used in industry as a gelling agent and as a prebiotic for the proper functioning of the intestinal flora (*18*).

It also contains polysaccharides (50 g/100 g), mainly cellulose and hemicellulose (*51*). The seed is rich in polyunsaturated and monounsaturated fatty acids, such as linoleic acid (51.7 %) and oleic acid (34.2 %), which are suitable for human consumption (*52*).

Several factors can influence the profile and amount of phytochemical compounds, including biotic and abiotic stress conditions, ultraviolet radiation, light intensity, fertilisation, water stress and the maturity of the plant (*53*).

The artichoke is characterised by bioactive compounds such as phenols and anthocyanins. Their highest mass fraction is found in the head and its edible parts (*5*, *54*), mainly in the form of monocaffeoylquinic and dicaffeoylquinic acids, as well as flavonoids derived from apigenin and luteolin (*20*). The leaves contain mainly phenolic acids, flavonoids and sesquiterpene lactones (*55*).

Phenolic compounds are the result of the secondary metabolism of cells, which are essential for growth and reproduction, and are produced under stressful conditions such as UV radiation, injury and infection (*56*). They are necessary for plant growth and reproduction, act as antipathogenic agents, and also contribute to pigmentation (*57*). Phenolic compounds have in their chemical structure an aromatic ring with one or more hydroxyl groups, including functional groups (*58*). Their structure is variable, and they are therefore multifunctional. About five thousand phenols are known, including flavonoids, simple phenols, phenolic acids, tannins, lignins, coumarins and tocopherols (*57*). They range from simple molecules to those with a high degree of polymerisation. In vegetables, they occur in a free form or in combination with proteins and sugars (glycosides) (*59*). In *Cynara,* the phenolic compounds belong mainly to the classes of caffeoylquinic acids and flavonoids (*60*).

The phenols of artichoke have the ability to modulate cellular antioxidants and several important enzymatic pathways (*3*). Thus, artichoke is able to reduce lipid peroxidation, reactive oxygen species (ROS) formation, protein oxidation and glutathione peroxidase (GSH-Px) activity (*61*, *62*).

**Fig. S1:**
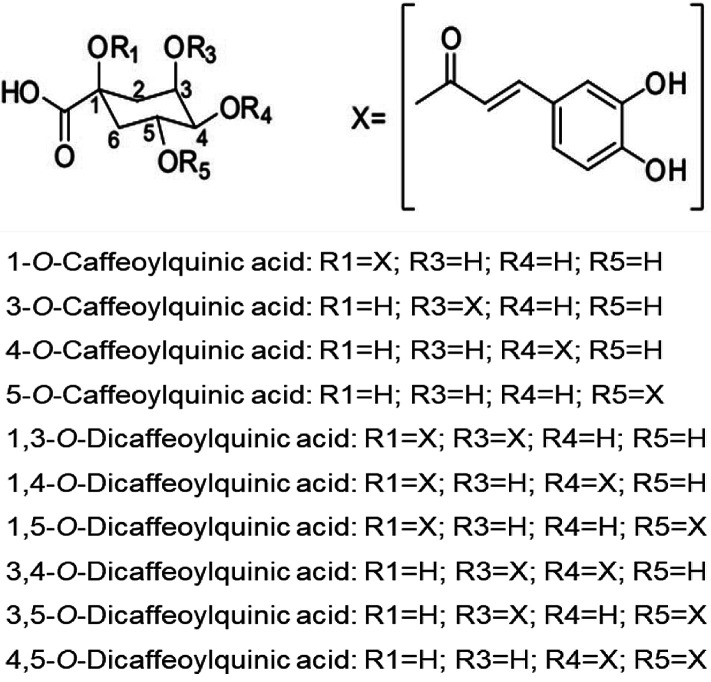
Chemical structures of caffeoylquinic acid derivatives in artichoke. Source: Adapted from Lattanzio *et al*. (*10*)

The most abundant phenols in artichoke flowers are those derived from caffeoylquinic acid, especially chlorogenic acid (5-*O*-caffeoylquinic acid), cynarin (1,3-*O*-dicaffeoylquinic acid), isochlorogenic acid (3,5-*O*-dicaffeoylquinic acid), flavonoids (apigenin and luteolin) and caffeoyl-glycoside and cyanidin derivatives (*3*, *4*, *63*). Fig. S1 shows the chemical structures of caffeoylquinic acid derivatives.

Chlorogenic acid and its isomers, the cryptochlorogenic, neochlorogenic and pseudochlorogenic acids, have several pharmacological activities, such as a chemopreventive effect in oncological diseases. In particular, chlorogenic acid can inhibit the formation of mutagenic compounds such as nitrosamines and the development of tumours (*53*). In addition, phenolic compounds have other health benefits such as: antithrombotic, antiallergic, anti-inflammatory, cardioprotective, antioxidant, antimicrobial and vasodilatory effects (*64*).

The content of polyphenolic compounds in artichokes is influenced by environmental factors, plant parts, genotype, agricultural management, postharvest practices (*53*, *65*) and extraction methods. The total mass fraction on dry mass basis of phenolic compounds (expressed as gallic acid equivalents (GAE)) in leaves, bracts, receptacles, flowers and stalks varies between 1191 and 3496 mg/100 g; flavonoids (expressed as quercetin equivalents (QE)) varies between 40.1 and 75.5 mg/100 g, and anthocyanins varies between 0.84 and 170.5 mg/100 g (Table 1 (*66*-*70*)).

**Table 1:** Methods for the extraction, operating conditions and quantification of bioactive compounds of artichoke

Part of artichoke	Extraction method/operating condition	Compound	*w*/(mg/100 g)	Reference
Leaf and stalk	*Mechanical agitation:* *m*(substrate):*V*(solvent)=1:20 (2 % formic acid in *V*(methanol):*V*(water)=4:1); homogenisation: 10 s vortex; agitation in horizontal shaker: 560 rpm, 15 min; centrifugation: 2647×*g*, 3 min	Phenolic compounds (as GAE on dry mass basis)	1191–3496	(*66*)
Bract and stalk	*Mechanical agitation:* *m*(substrate):*V*(solvent)=1:20 (75 % ethanol); agitation in horizontal shaker: 200 rpm, 60 °C, 40 min	Phenolic compounds (as GAE on dry mass basis)	2461	(*67*)
Head	*Mechanical agitation:* *m*(substrate):*V*(solvent)=1:10 (70 % methanol containing 1 mM of butylated hydroxytoluene and 1 mM hesperetin); agitation: 22 °C, 60 min; centrifugation: 2000×*g*, 3 min	Flavonoids (as QE on dry mass basis)	*Total luteolin:* Tondo di Paestum, Violetto di Sicilia and Cimiciusa: 2.5 *Total apigenin:* Cimiciusa: 747 Tondo di Paestum: 264 Violetto di Sicilia: 266	(*68*)
Bract and receptacle	*Maceration:* *m*(substrate):*V*(solvent)=1:5 (hexane); agitation: 3 h; filtration and drying; successive extractions: *m*(substrate):*V*(solvent)=1:5 (anhydrous diethyl ether, ethanol and water), 3 h	Flavonoids (as QE on dry mass basis)	Bracts: 75.5 Receptacles: 40.1	(*69*)
Flower	*Mechanical agitation:* *m*(substrate):*V*(solvent)=1:200 (70 % aqueous acetone and 0.01 % TFA); agitation: 4 °C, 1 h; centrifugation: 4000×*g*, 5 min; successive extractions: a) two extractions *m*(substrate):*V*(solvent)=1:200 (acetone), 45 min and 30 min; b) three extractions *m*(substrate):*V*(solvent)=1:100 (TFA and ethyl acetate, pH=1.5); centrifugation: 4000×*g*, 5 min	Anthocyanins (on dry mass basis)	Camus: 170 Green Globe: 87.8 Le Castel: 40.5 Petit Violet: 0.84 Buette: 104 Poivrade: 2.1	(*70*)

TFA=trifluoroacetic acid, GAE=gallic acid equivalents, QE=quercetin equivalents

The antioxidant, antimicrobial and cytotoxic activities of artichoke extracts are due to the presence of quercetin, catechin, chrysin, rosmarinic acid, apigenin and protocatechuic acid. The antimicrobial activity may be due to the permeabilisation of the cytoplasmic membrane, destabilisation and/or inhibition of enzymes (*71*–*73*). The mass fraction of rosmarinic acid in ethanolic extracts of bracts on dry mass basis is 1418 mg/100 g (*69*).

Noriega-Rodríguez *et al*. (*67*) reported a total mass fraction on dry mass basis of 2461 mg/100 g of phenolic compounds in artichoke waste consisting of the outer bracts and stalks. The edible parts of the artichoke may have a phenolic compound mass fraction ranging from 480 to 2980 mg/100 g (*74*).

Artichoke by-products consisting of petioles from residual shoots (branches from the root system of the plant) removed during thinning had 20835*–*27051 mg/kg of phenolic compounds as monocaffeoylquinic acids, dicaffeoylquinic acids and dicafeoyl succinylquinic acids (*66*).

According to Giménez *et al.* (*75*), tertiary artichoke heads have the highest mass fractionof phenolic compounds, followed by secondary and main heads. The determined total content of polyphenols in the edible fraction (inner bracts and receptacle) ranges from 5 to 12 mg/g on fresh mass basis.

Rocchetti *et al.* (*65*) found 365 compounds in fresh artichoke heads, with the highest number of flavonoids (144 compounds), 103 phenolic acids, followed by tyrosol, lignans and stilbenes with 62, 28 and 23 compounds, respectively. The by-products of the artichoke (stalks, leaves, secondary flowers and outer bracts) as well as the edible part contain large amounts of phenolic compounds, including caffeic acid and cynarin derivatives and flavones (luteolin, apigenin and their glycoside derivatives), which have been associated with beneficial health effects (*76*).

**Fig. S2:**
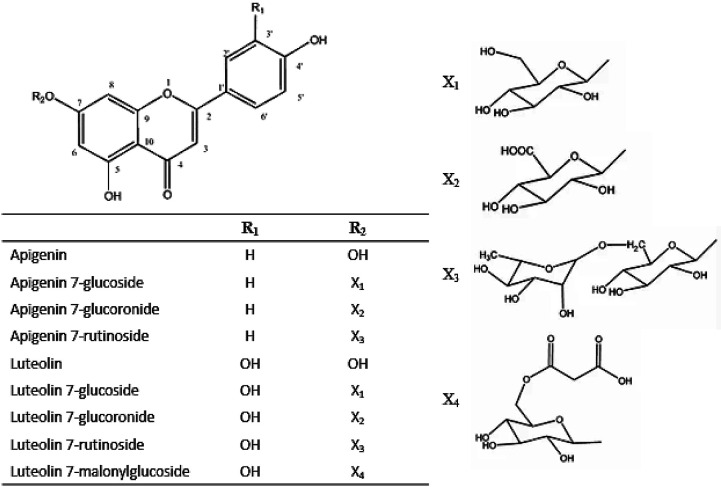
Chemical structures of the most important flavonoids and glycosides in artichoke. Source: Adapted from Lattanzio *et al*. (*10*)

An important class of natural polyphenols in artichokes are flavonoids, divided into subclasses such as flavones, flavonols, flavanones and flavanols. The most important flavones (Fig. S2) are apigenin, luteolin, apigenin-7-*O*-β-d-glucopyranoside, apigenin-7-*O*-rutinoside, luteolin-7-*O*-glucoside or cinnaroside and luteolin-7-rutinoside (*77*). Flavones and their glycosides are responsible for antioxidant (*78*) and anti-inflammatory properties (*79*), as well as lowering total cholesterol (LDL) and triglycerides (*80*). Ten flavonoids belonging to the flavone family (derived from apigenin and luteolin) have been identified and quantified in samples of Tudela artichoke (*81*).

The flavonoids in artichokes are mainly concentrated in the leaves and heads, and are absent in the flower stalk (*82*). Flavonoids have the ability to protect cells from oxidative damage. These compounds can be used as an antiatherosclerotic agent by decreasing the expression of monocyte chemoattractant protein-1 (MCP-1) (*83*). Luteolin and cinnarosides are responsible for these activities and they increase the expression of the endothelial nitric oxide synthase (eNOS) promoter and the mRNA of eNOS, leading to the production of nitric oxide and consequently to an antithrombotic and antiatherosclerotic effect (*84*).

The beneficial and health-promoting effects of artichoke and its use in the treatment of cardiovascular diseases are a consequence of these activities. The hypolipidaemic activity is due to the presence of luteolin, which blocks insulin-dependent 3-hidroxy-3-methylglutaryl-CoA reductase (HMG-CoA) activity, thus inhibiting cholesterol biosynthesis (*85*). Luteolin is a potential ingredient in skin photoprotection preparations against oxidative damage caused by ultraviolet A (UVA) radiation due to its lipophilic nature and the presence of *ortho*-dihydroxy groups (*86*). The total luteolin content is significantly higher in tertiary heads than in secondary or main heads. Genetic variability among cultivars influences the content of luteolin derivatives in floral capitulum orders (*87*).

Flavonoids may be able to inhibit bacterial growth by, among other things, altering the permeability of membranes and cell walls and inhibiting the synthesis of nucleic acids in Gram-positive and Gram-negative bacteria (*88*, *89*). The function of catechins as antioxidants is to bind ROS, limit free radicals such as superoxide, peroxyl, hydroxyl and DPPH (*90*, *91*) and also stimulate the expression of interleukin 10 (*92*). Catechins can increase the concentration of reactive oxygen species in cells and cause endogenous oxidative stress in the bacterium *Escherichia coli* by exerting an antibacterial effect by reducing the antioxidant potential of the bacterium (*93*). Catechins disrupt the cytoplasmic membrane of bacterial cells, preventing microbial growth of foodborne pathogens (*90*, *94*).

**Fig. S3:**
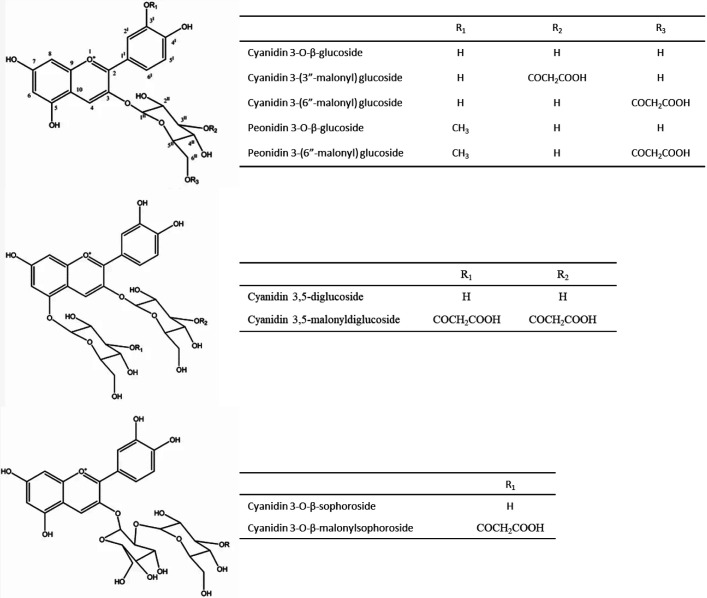
Chemical structure of anthocyanins and glycosides in artichoke. Source: Adapted from Lattanzio *et al*. (*10*)

Ethanol extracts from artichoke receptacles and bracts contain, expressed in quercetin equivalents (QE) per dry mass, about 40140 and 75540 mg/100 g, respectively. The predominant compounds in the extract of the bracts were catechin (1604 mg/100 g) and apigenin (526 mg/100 g), while the ethanolic extract of the receptacles contained 327 mg/100 g of chrysin (*69*). Another phenolic compound in the artichoke are anthocyanins (Fig. S3), which include cyanidin and its glycosides, peonidin and delphinidin glycosides (*70*). Some of these anthocyanins are non-acylated molecules with potent antioxidant activity (*95*–*97*). Acylation, especially with cinnamic acid derivatives, reduces the antioxidant and anti-inflammatory activity of anthocyanins (*95*).

Cyanidin downregulates the production of the inducible nitric oxide synthase (iNOS) isomer, which has a pro-inflammatory effect in the vascular system. Since eNOS and iNOS have different functions in the vascular system, the former is beneficial while the latter is harmful. Artichoke flavonoids, especially cynarin, activate the endothelial expression of NO (eNOS) with a vasoprotective effect, while the anthocyanins simultaneously decrease iNOS expression, indicating synergistic effects of the flavonoids. Artichoke has different effects against different forms of NOS, which contributes to its cardioprotective effect (*84*, *98*).

Schütz *et al.* (*70*) detected anthocyanins in the inner bracts of artichoke and identified cyanidin 3,5-diglucoside, cyanidin 3-glucoside, cyanidin 3,5-malonyldiglucoside, cyanidin 3-(3”-malonyl) glucoside and cyanidin 3-(6”-malonyl) glucoside. Cyanidin 3-(6''-malonyl) glucoside was the anthocyanin present in higher mass fraction in the violet petals. The violet petals of artichoke flowers contain on dry mass basis about 0.84 to 170 mg/100 g of anthocyanins.

According to Zazzali *et al.* (*99*), stalk had the highest anthocyanin mass fraction, measured as cyanidin-3-glucoside on fresh mass basis, among the artichoke parts, with about 0.76 mg/100 g, while the bracts also had a relatively high mass fraction of monomeric anthocyanins 0.19 mg/100 g.

### Enzymes

Artichoke and thistle (*C. cardunculus*) are similar varieties, and several enzymes from thistle have already been studied, including aspartic proteases such as cyprosins A, B and C and cardosins A, B, E, F, G and H, which have been isolated, purified and biochemically characterised (*100*, *101*). Aspartic proteases isolated under alkaline conditions are called cynarases or cyprosins (*102*, *103*), and proteases isolated at acidic pH from fresh stigmas of *C. cardunculus* are called cardosins (*14*, *104*, *105*). These proteolytic enzymes are used as natural coagulants (*3*).

Cardosin A accounts for 75*–*90 % of the total aspartic proteases in *C. cardunculus*, while cardosin B accounts for 10*–*25 % (*106*). Cardosins cleave bonds between amino acids with hydrophobic side chains and may have long hydrophobic sequences at the active site (*107*). Cardosin A, which consists of two peptides of 30 and 15 kDa (*14*), is similar to chymosin and has cleavage specificity (*108*). It cleaves bovine κ-casein between Phe105 and Met106 (*109*).

Cardosin B in *C. cardunculus* is a heterodimeric enzyme with an apparent molecular mass of 34 and 14 kDa and the amino acid sequence has 73 % similarity to cardosin A (*104*). It is similar to pepsin in terms of specificity and activity (*109*). It has greater proteolytic activity than cardosin A, but also less selective activity. It cleaves peptide bonds with at least one amino acid with aliphatic or aromatic side chains and other bonds with amino acids with basic side chains (*107*). These enzymes are more stable at pH=5.0 and temperatures below 45 °C and may be useful as coagulants in the production of some cheeses with soft and buttery textures and natural to slightly spicy flavours (*14*, *110*).

Cynarase A, B and C were isolated and purified from proteases of the glycoproteins of *C. cardunculus* var. *scolymus*, (*15*, *111*). Cynarase A consists of two subunits of approx. 32.5 and 16.5 kDa, cynarase B has two subunits of 33.5 and 16.5 kDa, and cynarase C consists of subunits of 35.5 and 13.5 kDa (*112*, *113*). Cynarase C has a higher specific activity than cynarases A and B, comparable to that of chymosin. Because of these properties, cynarases have proteolytic and milk clotting activity (*112*), with maximum activity at a pH of about 5.1 using casein as a substrate (*113*) and at pH=4.1 using synthetic peptides (*112*, *114*).

The artichoke contains an enzyme called polyphenol oxidase (EC 1.10.3.1), which is responsible for browning in plants. The main process responsible for quality loss during post-harvest storage is browning, together with peroxidase (EC 1.11.1.7), which is a limiting factor in artichoke processing. Therefore, artichoke can be considered a useful and cheap source of peroxidase and polyphenol oxidase of interest to industry. To date, only a few papers have been published on the purification and characterisation of artichoke peroxidase and polyphenol oxidase (*115*, *116*).

Table 2 (*14*, *15*, *111*, *115*-*117*) lists some methods for the extraction of artichoke (*C. cardunculus*) enzymes from different parts of the plant (leaves, flowers, stigmas, roots, stalks, receptacles and rhizomes) and the corresponding enzyme activities. The methods used for the extraction and/or separation of proteases, peroxidases and polyphenol oxidases included mechanical agitation, maceration, maceration combined with centrifugation and filtration, an ultrasonic system, and membrane filtration, showing that the enzymatic activity depends on the plant part and the method used.

**Table 2:** Methods of the extraction of artichoke enzymes, operating conditions and enzyme activity

Part of artichoke	Extraction method/operational condition	Enzyme	Activity	Reference
Leaf	*Mechanical agitation:* *m*(substrate):*V*(solvent)=1:3 (*c*(sodium citrate)=0.1 M, pH=3), time=3 min	Protease	Proteolytic: 13.2 U/mL Specific: 9.59 U/mg	(*117*)
	*Mechanical agitation:* *m*(substrate):*V*(solvent)=1:3 (sodium acetate 0.1 M, pH=5), time=3 min		Proteolytic: 7.9 U/mL Specific: 6.1 U/mg	
	*Mechanical agitation:* *m*(substrate):*V*(solvent)=1:3 (*c*(Tris-HCl)=0.1 M, pH=7), time=3 min		Proteolytic: 8.8 U/mL Specific: 6.6 U/mg	
	*Ultrasound:* *m*(substrate):*V*(solvent)=1:3 (*c*(sodium citrate)=0.1 M, pH=3); Operational conditions: 35 °C, 40 kHz, 60 min		Proteolytic: 14.4 U/mL Specific: 19.7 U/mg	
Flower	*Mechanical agitation:* *m*(substrate):*V*(solvent)=1:3 (*c*(sodium citrate)=0.1 M, pH=3); Centrifugation: 50000×*g*, 30 min	Cynarase A, B, C	Specific: Crude extract: 5 U/mg Cynarase A, B, C: 25, 100 and 20 U/mg	(*111*)
Flower and stigma	*Mechanical grind:* *m*(substrate):*V*(solvent)=1:8 (*c*(sodium citrate)=50 mM and c(NaCl)=1 M, pH=3); Centrifugation: 24 000 rpm, 20 min	Cynarase	Cynarase activity: 17.5 U/mL Cynarase specific activity: 6.5 U/mg	(*15*)
Leaf	*Maceration:* *m*(substrate):*V*(solvent)=1:10 (*c*(citric acid)=0.1 M, pH=3)	Aspartic protease	Specific proteolytic: 2.4 U/mg	(*14*)
Receptacle			Specific proteolytic: 1.5 U/mg	
Stalk			Specific proteolytic: 1.4 U/mg	
Flower			Specific proteolytic: 5.45 U/mg	
Head	*Mechanical grinding/Ultra-Turrax, speed 3:* *m*(substrate):*V*(solvent)=1.5:1 (*c*(phosphate)=0.1 M, pH=3), time=60 s; Centrifugation: 15 000×*g*, 30 min, 4 °C; Ultrafiltration: membrane 50 kDa	Polyphenol oxidase	Violetto di Sicilia (on fresh mass basis): 13.6–20 mmol/(min∙g)	(*115*)
			Violetto di Provenza (on fresh mass basis): 13–14.4 mmol/(min∙g)	
			Tema 2000 (on fresh mass basis): 19.3–27 mmol/(min∙g)	
Leaf	*Mechanical agitation:* *m*(substrate):*V*(solvent)=1:4 (*c*(sodium acetate)=0.05 M and 2 % polyvinylpyrrolidone, pH=6); Waring blender: 15 000 rpm, 3 min; Centrifugation: 17 400×*g*, 30 min, 4 °C	Peroxidase	Total activity: 12.1 U/mL Specific: 10 U/mg	(*116*)

Specific activity is expressed on protein mass basis

With respect to protease, the ultrasound-assisted extraction at 40 kHz, 35 °C, *m*(sample):*V*(solution)=1:3 (*c*(sodium citrate)=0.1 M, pH=3) and a time of 60 min gives the best enzymatic extraction of the leaf compared to other methods (mechanical agitation) and extraction conditions with other solutions (sodium acetate and Tris-HCl, pH=3, 5 and 7) (*117*). The flowers and stigmas are sources of cynarase (on protein mass basis 5-6.5 U/mg) (*15*, *111*), and mechanical agitation and mechanical grinding are methods that can be used for enzyme extraction. Aspartic protease can be extracted from various parts of the artichoke, with the highest specific proteolytic activities found in the flowers, followed by the leaves, receptacles and stalks of the inflorescence (*14*). Todaro *et al.* (*115*) investigated how harvest season affected the polyphenol oxidase activity and observed a decrease in activity in the spring harvest, with the exception of the cultivar Violettodi Provenza, which showed stable and low levels of enzymatic activity in both seasons. The variety Tema 2000 showed a decrease in activity during the spring harvest, but maintained the highest level of polyphenol oxidase activity. Cardinali *et al.* (*116*) extracted peroxidase by mechanical agitation and found that peroxidase from artichoke leaves on protein mass basis (10 Umg) was about 20 times higher than that extracted from the heads. This enzyme was shown to be stable at different temperatures (5 and 65 °C) and pH values (5.5–6.0).

Subsequent steps in the extraction of an enzyme include recovery, purification and concentration (*118*). The degree of purification of the enzyme depends on its use, especially for therapeutic or pharmaceutical purposes, and it is important that the enzyme is of high purity (*119*).

Various methods have been used to purify and/or concentrate enzymes, including membrane separation processes that act as a selective barrier, retaining particles larger than the pores while allowing smaller molecules to pass through the pores.

The classification of the processes is based on the type of force that drives the transport through the membrane (*120*) and is named according to the pore size: microfiltration, ultrafiltration, nanofiltration and reverse osmosis (*121*). For the purification and concentration of enzymes and proteins, ultrafiltration and nanofiltration are mainly used (*122*). Sidrach *et al*. (*15*) used ultrafiltration and nanofiltration to purify artichoke (*C. scolymus* L.) flower stigmas and obtained a specific activity of cynarase A on protein mass basis of 6.5 U/mg.

The aqueous two-phase method and precipitation using salts are also used in the separation of enzymes and proteins. Aqueous two-phase systems are ternary systems consisting of water and two non-volatile water-soluble components (*123*), which can be polymers, salts and/or ionic liquids (*124*). These systems can be used to separate proteins from cell debris or to purify proteins from other proteins. Soluble and particulate material concentrates in the lower, more polar phase, while proteins and enzymes concentrate in the upper, less polar and more hydrophobic phase (*125*).

Precipitation by means of salts is a very useful and practical method for the purification of proteins. The salt most commonly used in this procedure is ammonium sulphate, which is added as a solid or as a saturated solution to precipitate the proteins of interest (*126*). The precipitated proteins can be concentrated by removing the remaining ammonium sulphate solution and the protein pellet can then be dissolved in standard buffers or at low concentrations of ammonium sulphate (*120*). Pereira *et al*. (*123*) used for precipitation ammonium sulphate in the extract of stigmas and styles of dried artichoke flowers and obtained a proteolytic activity on protein mass basis of 0.24 and 0.79 ∆*A*/(g·min) and a coagulation activity of 90.6 and 74.6 UR/g at 30 and 70 % saturation, respectively.

### Applications of artichoke

Parts of the artichoke plant such as flowers, stems and leaves as well as residues from the processing of the bracts, can be (re)used in the food, chemical and pharmaceutical industries due to the bioactive compounds of the artichoke such as phenolic compounds, enzymes, soluble fibre, fructooligosaccharides, inulin and others. Table 3 (*6*, *16*, *79*, *85*, *127*-*144*) summarises the main uses of the artichoke plant and the functions in the different segments are described in detail below.

**Table 3:** Applications and functions of the artichoke in the food, chemical and pharmaceutical industries

Industrial application	Part of the artichoke	Compound and functions	Reference
Food and chemical	Inner bract	*In natura* and/or processed (chilled, frozen or preserved in brine)	(*6*)
	Flower	Aspartic proteases (EC 3.4.23) as vegetable coagulants, *e.g.* for cheese production	(*127*, *128*)
		Antioxidant (*e.g*. oil industry)	(*129*)
		Fructooligosaccharides and inulin	(*130*)
		Enzymes (polyphenol oxidase, peroxidase)	(*16*, *131*)
	Waste (leaves, flowers, stalks and others)	Cellulose and hemicellulose (paper pulp)	(*132*)
		Removal of phenols of wastewater	(*133*)
		Biofuel (bioethanol and biomethane)	(*134*–*136*)
		Adsorbent (*e.g*. treatment of effluents)	(*137*)
Pharmaceutical	Leaf Flower Stalk Bract	Enzymatic immunoassay	(*116*, *138*)
		Cardiovascular diseases and dyslipidaemia (reduction of LDL, total cholesterol and triglycerides)	(*85*, *139*, *140*)
		Anti-obesity	(*141*)
		Anticancer and chemopreventive	(*141*–*143*)
		Antioxidant and anti-inflammatory	(*79*, *144*)

### Chemical and food industry

The inner bracts and heart of the immature inflorescence (*3*, *4*, *17*, *35*) can be consumed *in natura* or processed chilled, frozen or preserved, with some ingredients added to the brine. For example, citric acid is used to lower the pH and prevent colour changes of the product, while calcium chloride is used to maintain the proper hardness of the vegetable during heat treatment (*145*).

Artichoke is used in industry because it is a potential source of various proteases (*127*, *128*). Artichoke flower extract is a promising vegetable coagulant that can add value to the cultivation of this species, as the discarded inflorescences, which are not marketed because of their size or appearance, can be used for protease extraction (*14*, *146*). The commercial enzymes used in milk coagulation are aspartic endopeptidase (EC 3.4.23), which are more active at acidic pH and are inhibited by pepstatin, and their amino acid sequences show high degree of homology (*129*, *130*, *147*, *148*). Artichoke flower also has an antioxidant capacity, which allows its use as an additive in the food industry to prevent the oxidation of oils (*129*).

By-products of artichoke flower processing can be used to extract fructooligosaccharides, inulin (*17*, *18*, *41*, *67*, *76*, *111*, *130*), polyphenol oxidase, peroxidase (*16*, *131*), flavonoids and phenolic compounds (*40*, *149*), and to isolate fractions rich in soluble fibre (*150*). Inulin from artichokes has been used as a fat substitute in chicken sausage recipes (*151*) and also in low-fat dairy products such as yoghurt (*152*).

Rodríguez-López *et al.* (*133*) have developed and patented a process that uses extracts from artichoke residues to catalyse the removal of phenols from aqueous solutions.

*C. cardunculus* is also used in industry as an energy crop to reduce greenhouse gas emissions and secure energy supply as it is involved in the production of solid biofuels by fermentation of lignocellulose and oil from cultivated and wild thistles (*153*). Biogas (bioethanol and biomethane) is a good example (*13*, *134*–*136*). To improve the yield of bioethanol production, new techniques have been applied to minimise the content of fermentation inhibitors. Additional pretreatment of artichoke residues is carried out during the process by autohydrolysis (water, *t*=121 °C) and acid hydrolysis at low acid content (0.5 % H_2_SO_4_, *t*=121 °C). These treatments promote a theoretical production of bioethanol per hectare of ~3900 kg (*8*).

The cellulose and hemicellulose from artichoke can be used for the production of paper pulp (*132*). Artichoke by-products have the potential to solve problems in the agro-food industry through the recovery of biological and inedible waste (leaves and stalks) and food additives (*27*, *28*, *154*), thus improving the recycling of waste from the processing of artichoke heads.

Chemically treated raw artichoke leaves can be used as a low adsorbent for the treatment of effluents from the textile industry and could be an alternative to the use of activated carbon (*137*). The peroxidase enzyme extracted from artichoke can also be used for the catalytic removal of phenolic and other aromatic compounds from wastewater. This method is a useful alternative for the treatment of industrial wastewater when conventional methods such as biological treatment are not available (*116*).

### Pharmaceutical industry

Artichoke extracts are used in the pharmaceutical industry for their potential medicinal properties. The extracts are usually obtained from the leaves of the artichoke plant (*C. scolymus*) and contain various bioactive compounds, including caffeoylquinic acids, flavonoids and cynaropicrin (*47*). These compounds are thought to contribute to the potential health benefits of artichoke extracts. One of the most important uses of artichoke extracts in the pharmaceutical industry is the treatment of digestive disorders. The extracts have traditionally been used as a herbal remedy for dyspepsia (indigestion), and research suggests that they may improve digestion by stimulating bile production and improving liver function (*64*). Artichoke extracts are often found in digestive supplements and may be beneficial for people with conditions such as irritable bowel syndrome or liver disease.

The use of artichoke leaf extracts has beneficial effects such as lowering serum levels of low-density lipoprotein (LDL), total cholesterol and triglycerides, without increasing levels of high-density lipoprotein (HDL), thus helping to treat disease (*85*). Cicero *et al*. (*138*) and Kraft (*139*) have studied the effect of the extract on LDL oxidation and cholesterol metabolism. Artichoke inhibits the biosynthesis of new hepatic cholesterol *via* inhibition of 3-hydroxy-3-methyl-glutaryl-CoA reductase and intestinal absorption of cholesterol and increases its excretion *via* inhibition of acetyl-coenzyme A acetyltransferase (ACAT). In addition, artichoke extracts have demonstrated their potential to support cardiovascular health. Studies have shown that the bioactive compounds in artichoke extracts, particularly cynaropicrin, can help lower cholesterol levels by inhibiting the enzyme involved in cholesterol synthesis. By lowering cholesterol levels, artichoke extracts have the potential to support heart health and reduce the risk of cardiovascular disease.

The anti-obesity effect is due to several mechanisms, such as the inhibition of digestive enzymes (lipase, α-glucosidase and α-amylase), while bile secretion, lipolysis and lipid metabolism are stimulated. Inhibition of the secretion of inflammatory adipokines is also associated with the anti-obesity and prebiotic effects of inulin (*140*).

There are studies on possible anticancer and chemopreventive activities against various cancer cell lines, including caspase-independent mechanisms in the inhibition of breast cancer *via* induction of senescence-associated β-galactosidase (SA-β-gal) and upregulation of tumour suppressor genes (*141*), apoptosis of the mesothelioma cell line (*142*) and human hepatoma cells (*143*).

The antioxidant and anti-inflammatory effects are due to the presence of polyphenols (*79*). Studies of antioxidant activity *in vitro* are conducted with DPPH (1,1-diphenyl-2-picrylhydrazyl), ABTS (2,2-azino-bis-3-(ethylbenzothiazoline-6-sulfonic acid)), FRAP (iron(III) reducing antioxidant power) and β-carotene bleaching assays (*7*, *144*). *In vivo* effects were determined using oxidative stress biomarkers such as superoxide dismutase-1 (SOD), catalase (CAT) and reduced glutathione (GSH) for antioxidant activity and inhibition of carrageenan-induced oedema activities, as well as histopathological studies (*79*). In *in vitro* studies, normal cell lines were exposed to inflammatory cytokines, hydrogen peroxide and ultraviolet B (UVB), confirming the effect of the leaves in reducing the production of harmful and destructive reactive oxygen species (ROS) in cells. *In vivo* studies and meta-analyses confirmed the antioxidant effect of artichoke extract in animals, mediated by the increase of liver-protective enzymes against free radicals (CAT, SOD and GSH-Px) and the reduction of malondialdehyde content in the liver and plasma (*29*).

The hepatoprotective effect is due to its antioxidant stress effect and inhibition of Toll-like receptor I (TLR4) and its signalling pathway and nuclear factor-kappa B (NF-κB) or TLR4/NF-κB (*155*), regenerating dysfunctional liver cells (*156*) and healing the regulatory mechanism, allowing repair of DNA damage after hepatotoxicity (*157*). This effect is evidenced by the reduction of alanine aminotransferase (ALT) and aspartate aminotransferase (AST) content in serum.

In addition, artichoke has an enzyme such as peroxidase that has attracted industry attention because of its purified usefulness as a catalyst in clinical biochemistry and enzymatic immunoassays (*116*).

It is important to note that while artichoke extracts show promise in these areas, further research is needed to fully understand their mechanisms of action and evaluate their efficacy and safety. As with any pharmaceutical product or food supplement, it is important to consult a healthcare professional before using artichoke extracts for medicinal purposes.

## CONCLUSIONS

This review provides information on the phytochemical composition, bioactivity and possible uses of artichoke. There is evidence of applications in the food industry with preserved and/or frozen bracts, food additives, inulin in the preparation of low-fat foods and as a prebiotic, soluble fibre and fructooligosaccharides as prebiotics, proteolytic enzymes as natural coagulants and phenolic compounds with antioxidant activity to prevent the autoxidation of lipids in foods and/or oils. In addition, they can be used in other industrial applications such as biofuels, pulp production (cellulose and hemicellulose) and others. Due to their bioactive properties (including antioxidant, anti-inflammatory, anticancer, hypocholesterolaemic and cardioprotective effects), they can be used as bioactive additives for nutraceutical purposes.
